# Transcriptomic Signature of 3D Hierarchical Porous Chip Enriched Exosomes for Early Detection and Progression Monitoring of Hepatocellular Carcinoma

**DOI:** 10.1002/advs.202305204

**Published:** 2024-02-07

**Authors:** Kezhen Yi, Yike Wang, Yuan Rong, Yiru Bao, Yingxue Liang, Yiyi Chen, Fusheng Liu, Shikun Zhang, Yuan He, Weihuang Liu, Chengliang Zhu, Long Wu, Jin Peng, Hao Chen, Weihua Huang, Yufeng Yuan, Min Xie, Fubing Wang

**Affiliations:** ^1^ Department of Laboratory Medicine Zhongnan Hospital of Wuhan University No.169 Donghu Road, Wuchang District Wuhan 430071 P. R. China; ^2^ College of Chemistry and Molecular Sciences Wuhan University Wuhan 430072 P. R. China; ^3^ Department of Hepatobiliary & Pancreatic Surgery Zhongnan Hospital of Wuhan University Wuhan Hubei 430071 P.R. China; ^4^ Medical Research Center for Structural Biology School of Basic Medical Sciences Wuhan University Wuhan 430072 P. R. China; ^5^ Department of Clinical Laboratory Institute of Translational Medicine Renmin Hospital of Wuhan University Wuhan Hubei 430060 P. R. China; ^6^ Department of Oncology Renmin Hospital of Wuhan University Wuhan 430060 P. R. China; ^7^ Department of Radiation and Medical Oncology Zhongnan Hospital Wuhan University Wuhan 430071 P. R. China; ^8^ Department of Pathology Zhongnan Hospital of Wuhan University Wuhan 430071 P. R. China; ^9^ Clinical Medicine Research Center for Minimally Invasive Procedure of Hepatobiliary & Pancreatic Diseases of Hubei Province Wuhan Hubei 430071 P. R. China; ^10^ Tai Kang Center for Life and Medical Sciences Wuhan University Wuhan Hubei 430071 P. R. China; ^11^ Center for Single‐Cell Omics and Tumor Liquid Biopsy Zhongnan Hospital of Wuhan University Wuhan 430071 P. R. China; ^12^ Wuhan Research Center for Infectious Diseases and Cancer Chinese Academy of Medical Sciences Wuhan 430071 P. R. China

**Keywords:** exosome, hepatocellular carcinoma, liquid biopsy, long noncoding RNA, microfluidic chips

## Abstract

Hepatocellular carcinoma (HCC) is a highly lethal malignant tumor, and the current non‐invasive diagnosis method based on serum markers, such as α‐fetoprotein (AFP), and des‐γ‐carboxy‐prothrombin (DCP), has limited efficacy in detecting it. Therefore, there is a critical need to develop novel biomarkers for HCC. Recent studies have highlighted the potential of exosomes as biomarkers. To enhance exosome enrichment, a silicon dioxide (SiO_2_) microsphere‐coated three‐dimensional (3D) hierarchical porous chip, named a SiO_2_‐chip is designed. The features of the chip, including its continuous porous 3D scaffold, large surface area, and nanopores between the SiO_2_ microspheres, synergistically improved the exosome capture efficiency. Exosomes from both non‐HCC and HCC subjects are enriched using an SiO_2_‐chip and performed RNA sequencing to identify HCC‐related long non‐coding RNAs (lncRNAs) in the exosomes. This study analysis reveales that LUCAT‐1 and EGFR‐AS‐1 are two HCC‐related lncRNAs. To further detect dual lncRNAs in exosomes, quantitative real time polymerase chain reaction (qRT‐PCR) is employed. The integration of dual lncRNAs with AFP and DCP significantly improves the diagnostic accuracy. Furthermore, the integration of dual lncRNAs with DCP effectively monitors the prognosis of patients with HCC and detects disease progression. In this study, a liquid biopsy‐based approach for noninvasive and reliable HCC detection is developed.

## Graphical abstract

Hepatocellular carcinoma (HCC), a deadly tumor, lacks effective non‐invasive diagnosis via current serum markers. The innovative SiO_2_‐chip enriches exosomes efficiently, unveiling HCC‐specific lncRNAs like LUCAT‐1 and EGFR‐AS‐1. Validated by qRT‐PCR, integrating these lncRNAs with traditional markers significantly enhances diagnostic accuracy. Moreover, their combination with DCP aids in monitoring HCC progression. This liquid biopsy approach offers reliable non‐invasive HCC detection.

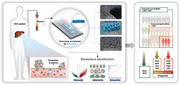

## 1 Introduction

Primary liver cancer, with HCC as the predominant form, is prevalent worldwide.^[^ ^1^, ^2^ ^]^ However, the diagnostic accuracy of imaging analysis in clinical applications remains low, primarily due to its reliance on the subjective experience of physicians.^[^ ^3^, ^4^ ^]^ Similarly, existing diagnostic models (e.g., Okuda, TNM, BCLC, JIS, CUPI, CLIP, GRETCH, CIS) primarily depend on clinical information, limiting their ability to offer precise prognostic predictions.^[^ ^5^, ^6^ ^]^ As a result, there is growing recognition of the significance of early management in improving long‐term survival outcomes for HCC. This has spurred an urgent demand for a robust molecular biomarker‐based approach capable of effectively diagnosing and monitoring patients, thereby providing essential guidance for treatment strategies.^[^ ^7^ ^]^

Several serum‐based biomarkers, including bilirubin, albumin, AFP‐L3, AFP, and DCP, have been used for the diagnosis of HCC.^[^ ^8^, ^9^ ^]^ However, their sensitivities and specificities are not ideal. For instance, AFP is commonly used as a serum biomarker in the management of patients.^[^ ^10^ ^]^ However, it is important to note that approximately 30% of patients with HCC are AFP‐negative.^[^ ^11^ ^]^ Therefore, there is an urgent need to develop reliable and novel noninvasive biomarkers that can accurately diagnose and predict the prognosis of HCC.

The widespread utilization of next‐generation sequencing has greatly advanced the identification of blood biomarkers, including proteins, deoxyribonucleic acids (DNAs), and ribonucleic acids (RNAs), using a method known as liquid biopsy.^[^ ^12^, ^13^, ^14^ ^]^ LncRNAs in the transcriptome are characterized by a length of more than 200 bp. Dysregulation of lncRNAs has been observed in various cancers, including HCC.^[^ ^15^, ^16^, ^17^ ^]^ Unlike messenger RNAs, lncRNAs are functional molecules and their expression levels provide a better reflection of disease status. The highly specific expression patterns of lncRNAs make them valuable for precise staging or classification of certain diseases.^[^ ^18^ ^]^ Notably, the expression patterns of lncRNAs in exosomes vary across different cells and under various physiological and pathological conditions, suggesting the potential of exosomal lncRNAs as diagnostic tools.^[^ ^19^, ^20^ ^]^ However, current exosome enrichment technology suffers from low efficiency, and the expression levels of lncRNAs are low, posing limitations to downstream analyses.^[^ ^21^, ^22^ ^]^ Thus, the development of an effective exosome enrichment strategy is crucial for overcoming these challenges.

Ultracentrifugation is currently considered the “gold standard” for exosome isolation. However, its application in clinical analysis is challenging because of the requirement for large sample volumes and complex operating flows.^[^ ^23^ ^]^ Compared to traditional methods, microfluidic technology has the advantages of easy manipulation, convenient integration, precise control of complex fluids, low sample consumption, and cost‐effectiveness.^[^ ^24^, ^25^, ^26^ ^]^ Thus, numerous microfluidic‐based methods have been employed and recognized as valuable tools for enriching exosomes by taking advantages of their physical properties and biological signatures.^[^ ^27^, ^28^ ^]^ Generally, size‐based exosome isolation methods are beneficial for maintaining the natural state of exosomes; however, the purity of the isolated exosomes is still influenced by proteins and other impurities, limiting downstream analyses. Immunoaffinity‐based microfluidic chips are widely used for rapid and specific separation of high purity exosomes. However, these chips require delicate and complex designs, which restrict their large‐scale clinical applications. Therefore, it is desirable to develop a novel microfluidic chip for the facile and efficient isolation and downstream analysis of exosomes.

In this study, we successfully developed a SiO_2_ microsphere‐coated 3D hierarchical porous chip, named SiO_2_‐chip, for the enrichment of exosomes. The interconnected micropores of the 3D scaffold induced a chaotic fluid flow, and the SiO_2_ microspheres significantly reduced the pore size of the scaffold, providing a large surface area and reducing the fluid boundary effects. Our method exhibited outstanding performance by directly enriching exosomes derived from plasma, achieving an impressive detection limit as low as 10000 particles mL^−1^. It is worth highlighting that the entire analysis process demanded a minimal volume of 40 µL of plasma and could be completed rapidly within a 10‐min timeframe. Using this innovative chip, we collected plasma exosomes from patients as well as appropriate control subjects. Subsequently, we conducted a comprehensive genome‐wide transcriptome analysis to identify the novel features of lncRNAs in HCC exosomes. With the discovery of biomarkers, we rigorously assessed and validated the performance of this non‐invasive circulating feature in clinical cohorts to evaluate its diagnostic and prognostic capabilities in patients with HCC.

## 2 Results

### 2.1 Optimization and Characterization of the SiO_2_ Microsphere‐Coated 3D Porous Scaffold

**Figure 1:**
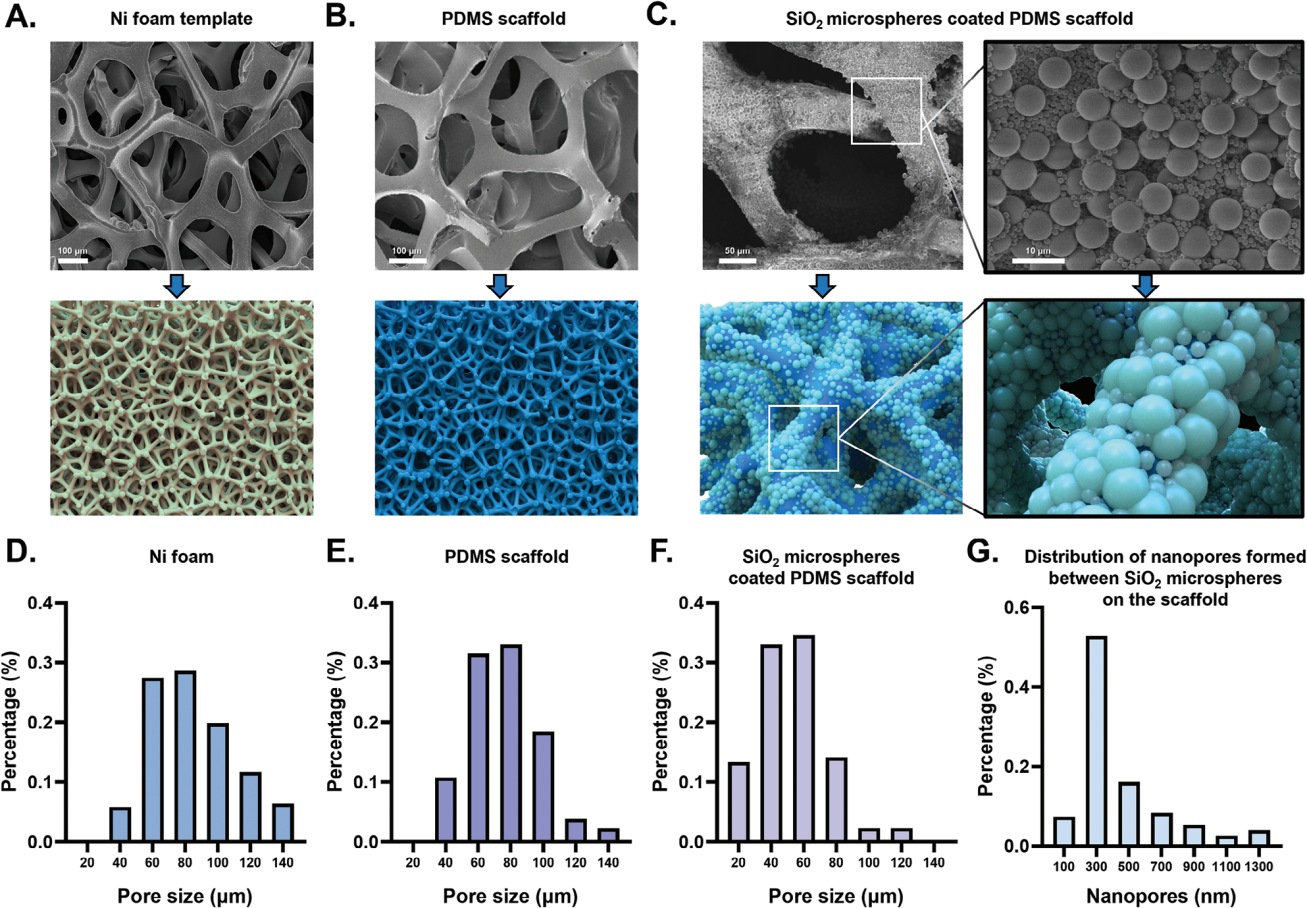
The construction of the SiO_2_ microspheres coated 3D PDMS scaffold. A) Scanning electron microscopy (SEM) image and diagrammatic drawing of Ni foam template B) SEM image and diagrammatic drawing of the prepared PDMS scaffold C) SEM image and diagrammatic drawing of the SiO_2_ microsphere‐coated PDMS scaffold (denoted as SiO_2_/PDMS scaffold). The enlarged pictures on the right show the details. D‐F) Pore size distribution of the Ni foam D), PDMS scaffold E) and the SiO_2_/PDMS scaffold F). G) The distribution of nanopores formed between stacked SiO_2_ microspheres on the scaffold. Abbreviations: PDMS (Polydimethylsiloxane), SiO_2_ (Silicon dioxide).

Based on previous studies, we utilized a sacrificial template method to fabricate a 3D polydimethylsiloxane (PDMS) scaffold.^[^ ^28^, ^29^ ^]^ Initially, small‐aperture Ni foam was packed as a template with uncross‐linked PDMS via high‐speed centrifugation. Subsequently, the excess PDMS was removed from the pores of the Ni foam by low‐speed secondary centrifugation, leaving only a surface‐covered PDMS layer. The effects of different speeds (3750, 4250, and 5000 rpm) during secondary centrifugation on the resulting 3D scaffold were investigated. Notably, at 4250 rpm, the prepared 3D PDMS scaffold had no significant dead pores and remained relatively complete and uniform (Figure S1). Consequently, 4250 rpm was chosen as the secondary centrifugation speed for fabricating the PDMS scaffold. As shown in **Figure** **1**A,B,D,E, the prepared PDMS scaffold displayed continuous pores with a pore size distribution ranging from to 40–140 µm, which was similar to the Ni foam template used in the fabrication process. These findings provide clear evidence of the successful replication of the porous structure of Ni foam in the resulting 3D PDMS scaffold.

Compared to the laminar flow mode observed in traditional straight‐line channels, the pore structure of 3D porous materials enables fluid turbulence within the channel, thereby increasing the contact frequency between the fluid and capture substrate. Then, the mixed 1 and 5 µm carboxylated silicon dioxide microspheres (SiO_2_ microspheres) were modified simultaneously on the surface of PDMS scaffold by electrostatic adsorption and layer‐by‐layer self‐assembly method (denoted as SiO_2_/PDMS scaffold). We investigated the effect of modified SiO_2_ microspheres on the morphology and pore size of the scaffold. Figure 1C shows that when SiO_2_ microspheres of different sizes were mixed and co‐assembled, the microspheres were uniformly and closely packed on the surface of the scaffold. Furthermore, the pore size of SiO_2_/PDMS scaffold was obviously reduced and the distribution was concentrated in the range of 20–80 µm (Figure 1F). By scanning electron microscope (SEM) image at high minification, we discovered the presence of nanopores between the stacked 1 and 5 µm SiO_2_ microspheres in the scaffold. After statistical analysis, we found that when 1 and 5 µm SiO_2_ microspheres were mixed and co‐assembled on the scaffold, the size distribution of these nanopores showed a wide and suitable range from tens to over a thousand nanometers. A substantial proportion of these nanopores was smaller than 500 nm (Figure 1G and S2). These results show that the mixture modification of multilayer microspheres of different sizes effectively reduced the pore size of the scaffold, providing a large specific surface area and a wide range of nanopores on the scaffold surface. This may contribute to enhanced fluid turbulence within the channel, increased antibody immobilization on the scaffold surface, and reduced fluid boundary effects, thereby synergistically improving the exosome capture efficiency.

Overall, the constructed SiO_2_/PDMS scaffold had several advantages. The 3D porous scaffold features a continuous interconnected porous structure that transforms the fluid flow within the channel from laminar to turbulent, thereby increasing the collision frequency between the exosomes and substrate. By assembling the SiO_2_ microspheres on the scaffold, the pore size of the scaffold was effectively reduced, whereas the specific surface area within the channel increased. SiO_2_ microspheres of varying sizes create nanoscale gaps, minimizing the boundary effect and enhancing the binding between the exosomes and the scaffold surface. These synergistic effects might serve to significantly improve the capture efficiency of the exosomes.

### 2.2 High‐Efficiency Exosome Enrichment Through Functionalized SiO_2_‐Chip

To achieve efficient exosome enrichment from the plasma, the SiO_2_/PDMS scaffold was meticulously functionalized through a sequential process involving streptavidin (SA) and biotinylated anti‐CD63 antibody immobilization. The antibody‐functionalized SiO_2_/PDMS scaffold was then integrated into a customized single‐channel manifold for the microchip platform, which was called as SiO_2_‐chip.^[^ ^30^, ^31^ ^]^

**Figure 2:**
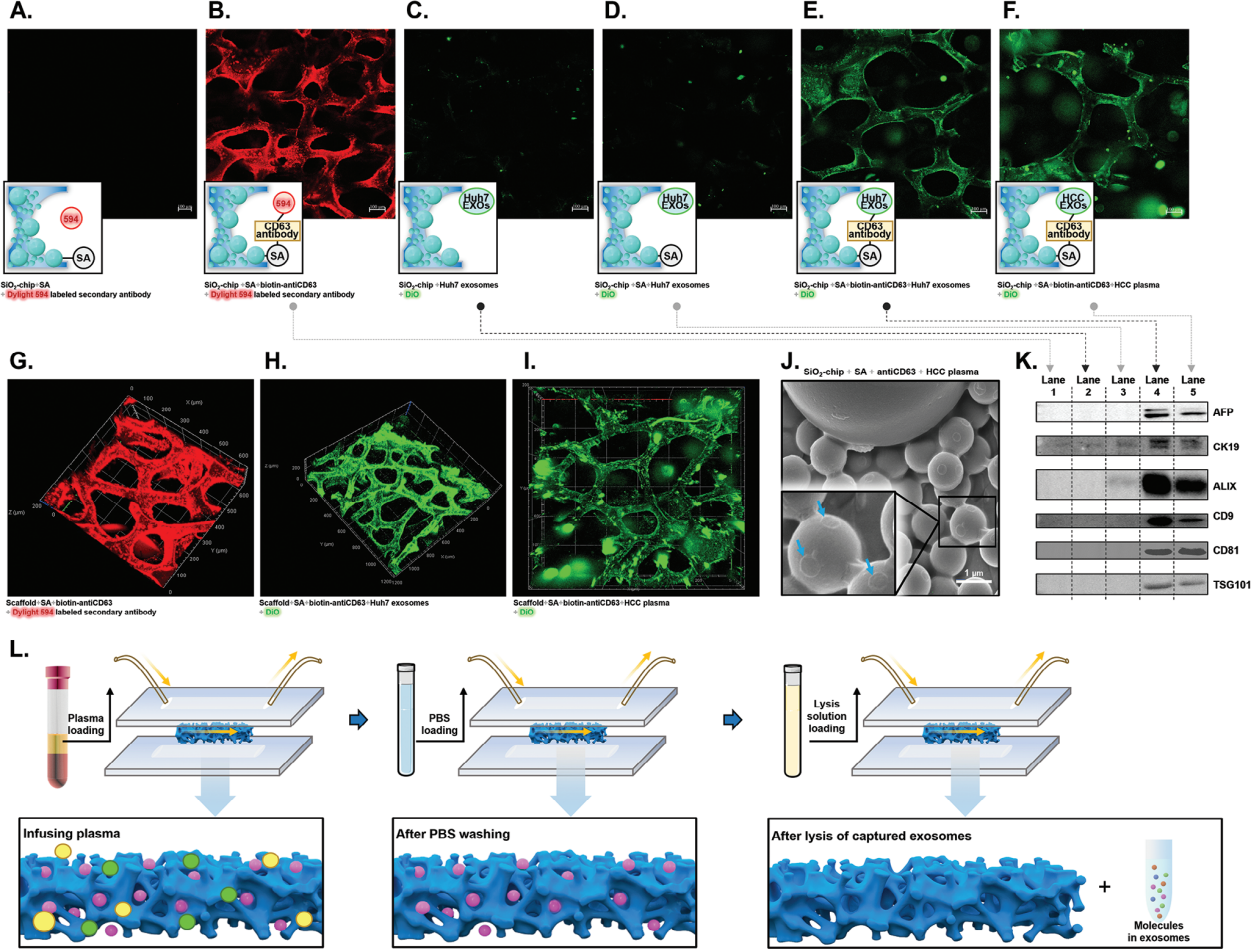
Characterization of exosome enrichment performance using the functionalized SiO_2_‐chip. A–F) Fluorescence imaging of the SiO_2_‐chip, including a simulation in the bottom‐left. Details of experimental grouping are provided in the text under figures. G–I) 3D views of the chip. Details of experimental grouping are provided in the text under figures. J) SEM image of exosomes captured on the functionalized SiO_2_‐chip K) Western blot (WB) imaging of exosomes captured by differently SiO_2_‐chip, where lane 1–5 correspond to different modification methods. Grouping for lane 1–5 is consistent with B–F. L) The exosomes enrichment process of the SiO_2_‐chip. Plasma was introduced into the chip and subsequently washed with PBS solution to eliminate impurities, leaving only the captured exosomes on the SiO_2_‐chip. RNA lysates or RIPA lysates were passed through the chip to extract RNA or proteins from exosomes. Abbreviations: SA (streptavidin), biotin‐anti CD63 (biotinylated anti‐CD63 antibody), EXO (exosome), HCC (hepatocellular carcinoma), and PBS (phosphate‐buffered saline). Unveiling HCC‐associated lncRNAs in exosomes enriched by SiO_2_‐chip through transcriptome sequencing

To validate the successful binding of CD63 antibodies to the SiO_2_‐chip, the modified SiO_2_‐chip was incubated with DyLight 594‐labeled secondary antibodies and examined by confocal microscopy.   As shown in **Figure** **2**A and B, the anti‐CD63 antibody‐modified SiO_2_‐chip showed strong red fluorescence, while, SiO_2_‐chip without modification with antibody had no fluorescence. The 3D image of the functionalized SiO_2_‐chip was presented in Figure 2G. These results provided definitive evidence for the successful attachment of CD63 antibodies to the SiO_2_‐chip.

Following ultracentrifugation, exosomes derived from the Huh7 cell line were introduced into the anti‐CD63 antibody‐modified SiO_2_‐chip and subsequently subjected with PBS to remove impurities. The characteristics of huh‐7 derived exosomes were shown in Figure S3 (Supporting Information). Exosomes were stained with DIO and visualized by confocal microscopy. Notably, Figure 2E displayed a vivid green fluorescence, in contrast to Figure 2C,D, indicating the efficient capture of exosomes from the Huh7 cell supernatant by the anti‐CD63 antibody‐modified SiO_2_‐chip. The 3D fluorescence results of the functionalized SiO_2_‐chip after introduction of exosomes from the cell supernatant are shown in Figure 2H.

Furthermore, the functionalized SiO_2_‐chip was evaluated for direct use with clinical samples by introducing plasma from patients with HCC. The fluorescence staining results obtained by confocal microscopy demonstrated the capability of the functionalized chip to enrich plasma exosomes (Figure 2F). Figure 2I depicted the 3D fluorescence results of the functionalized SiO_2_‐chip after the introduction of the HCC plasma.

Scanning electron microscopy (SEM) revealed the presence of cup‐shaped vesicles on the surface of the functionalized SiO_2_‐chip, confirming the successful capture of exosomes from the plasma (Figure 2J). Based on the SEM imaging results after the capture of exosomes on the chip, it was clear that the captured exosomes exhibited a broad size distribution ranging from 50 to 220 nm, with an average size of approximately 100 nm (Figure S4, Supporting Information).

Western blot imaging provided additional confirmation of the enhanced enrichment effect achieved by the functionalized SiO_2_‐chip on exosomal biomarkers. These included transmembrane proteins (CD9, CD81), membrane‐associated proteins (TSG101, ALIX), and proteins associated with HCC (AFP, CK19) (Figure 2K).

Collectively, these results substantiate the effectiveness of the functionalized SiO_2_‐chip for efficient exosome capture and highlight the potential of this method for direct exosome enrichment in clinical plasma samples.

### 2.3 Unveiling HCC‐Associated lncRNAs in Exosomes Enriched by SiO_2_‐Chip Through Transcriptome Sequencing

**Figure 3:**
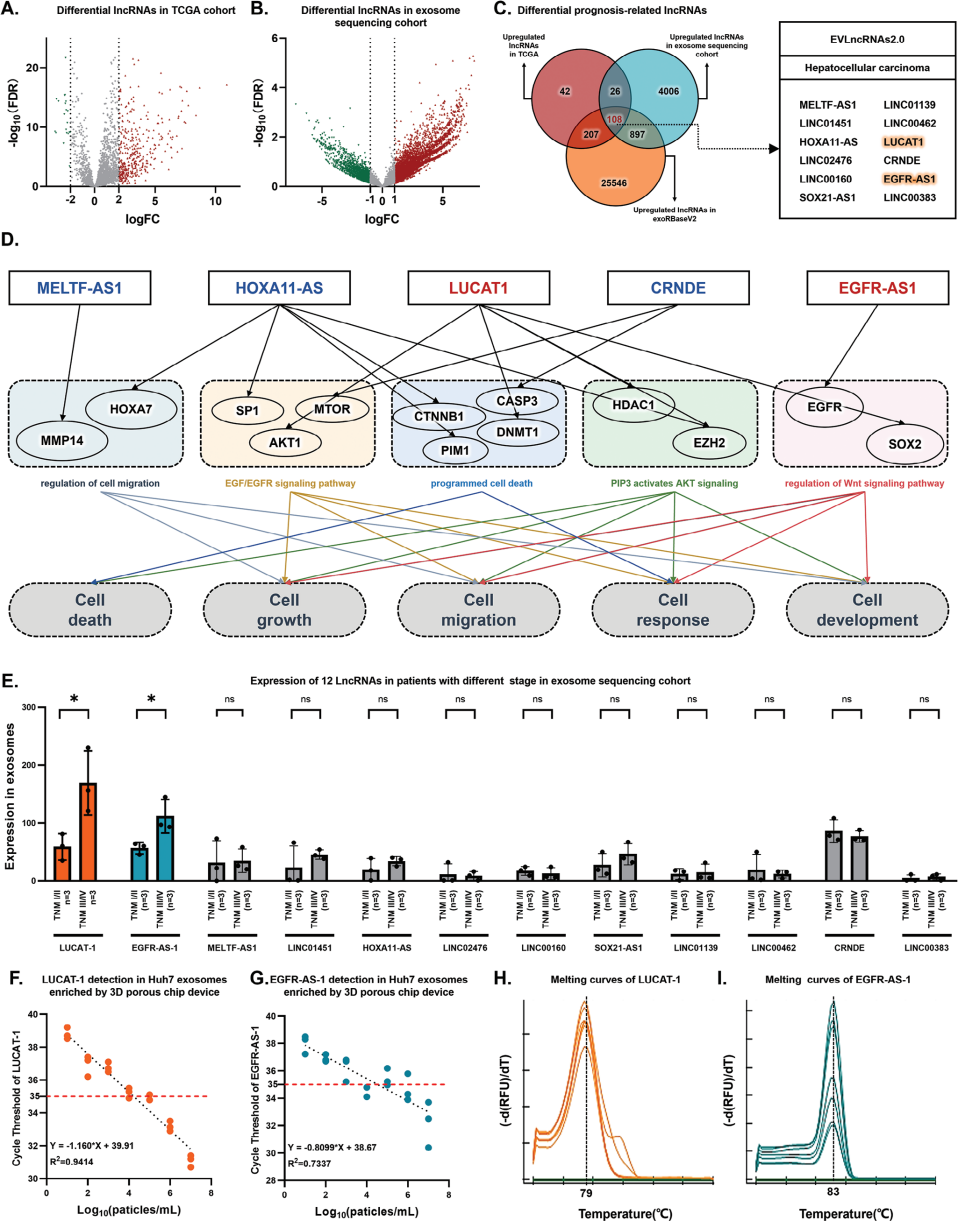
Identification of prognosis‐related biomarkers in plasma exosomes of HCC patients. A) Volcano plot showing differential LncRNAs in plasma exosomes of HCC patients versus healthy control samples (TCGA cohort). B) Volcano plot showing prognosis‐related LncRNAs in HCC tissue versus healthy control tissues (ES cohort). C) Venn diagram demonstrating the 108 differential LncRNAs. Out of 108 LncRNAs, 12 have been validated in the EVLncRNAs2.0 database as being associated with HCC. D) LncRNAs–protein pathway function network. E) Expression level of 12 LncRNAs in plasma exosomes of HCC patients with different TNM stage (ES cohort), presented as mean ± SD, with significance assessed using a *t*‐test. F) Sensitivity and linear range of LUCAT‐1 detecting in Huh7 exosomes enriched by functionalized SiO_2_‐chip (n = 3 independent technical replicates). R^2^, coefficient of determination. G) Sensitivity and linear range of EGFR‐AS‐1 detecting in Huh7 exosomes enriched by functionalized SiO_2_‐chip (n = 3 independent technical replicates). R^2^, coefficient of determination. H) Melting curves of LUCAT‐1 detecting in Huh7 exosomes enriched by 3D functionalized SiO_2_‐chip. I) Melting curves of EGFR‐AS‐1 detecting in Huh7 exosomes enriched by functionalized SiO_2_‐chip. Abbreviations: TCGA (The Cancer Genome Atlas) and ES cohort (exosome sequencing cohorts).

To identify molecular markers that can distinguish HCC from healthy individuals and high‐risk disease, we collected plasma exosomes from six individuals diagnosed with HCC and six non‐HCC subjects, including two healthy individuals, two hepatitis B virus (HBV)‐infected individuals, and 2 liver cirrhosis (LC), using a functionalized SiO_2_‐chip. This cohort was termed the Exosome Sequencing Cohort (ES Cohort). Transcriptome sequencing of exosomes from this cohort revealed 5036 upregulated lncRNAs. To further discover molecular markers associated with HCC prognosis, we performed differential gene analysis based on The Cancer Genome Atlas (TCGA) data, identifying 392 upregulated lncRNAs. To confirm that these lncRNAs were highly expressed in the exosomes, we obtained a list of highly expressed lncRNAs (19 442 upregulated lncRNAs) from the exoRBaseV2 database. Considering the intersection of these three sets, we identified 108 overlapping genes, indicating their potential importance in HCC (**Figure** **3**A–C). The screened genes are listed in Table S1 (Supporting Information).

To ensure their potential for clinical application, we further screened these 108 genes in the EVLncRNAs2.0 database, a comprehensive resource for experimental verification of functional lncRNAs. We selected 12 genes: MELTF‐AS1, LINC01451, HOXA11‐AS, LINC02476, LINC00160, SOX21‐AS1, LINC01139, LINC00462, LUCAT‐1, CRNDE, EGFR‐AS1, and LINC00383.

Regulatory networks were constructed for the target genes of these 12 lncRNAs and we obtained results for five of them: MELTF‐AS1, HOXA11‐AS, LUCAT‐1, CRNDE, and EGFR‐AS1. These findings shed light on the roles of the identified lncRNAs in HCC progression. As shown in Figure 3D, the regulatory networks involved multiple key pathways related to tumor growth, progression, and migration. Target genes and their functional predictions are shown in Tables S2 and S3 (Supporting Information).

To enhance the reliability of the selected molecular markers, we examined the expression levels of these 12 genes at different stages in the ES Cohort. We observed that LUCAT‐1 and EGFR‐AS1 were significantly upregulated in HCC patients with advanced stages compared to those with earlier stages. These findings further support the potential utility of lncRNAs as promising markers for HCC diagnosis and prognosis.

Figure S5 (Supporting Information) shows the detailed process of selecting HCC‐associated lncRNAs in exosomes. Based on these findings, our next step will be to investigate whether the identified lncRNAs, LUCAT‐1 and EGFR‐AS1, can be detected in exosomes enriched using the functionalized SiO_2_‐chip using clinically available qPCR methods. In addition, we explored the clinical applications of these two lncRNAs in the management of HCC.

After identifying prognosis‐associated lncRNAs in HCC through transcriptome sequencing of exosomes enriched with a functionalized SiO_2_‐chip, we investigated the potential of using functionalized SiO_2_‐chip‐enriched exosomes for qRT‐PCR ‐based detection of LUCAT‐1 and EGFR‐AS‐1. Initially, we analyzed Huh7 cell‐derived exosomes at various concentrations to determine the sensitivity of qRT‐PCR detection using exosomes enriched with functionalized SiO_2_‐chip. The results demonstrated a gradual decrease in the cycle threshold (ct) as the concentration of Huh7‐derived exosomes increased from 10 to 10 000 000 particles µL^−1^ (Figure 3F,G). For qRT‐PCR analysis, a CT value not obtained or exceeding 40 was considered negative, whereas a CT value <35 was considered positive. Based on this criterion, the detection limit for lncRNAs in Huh7‐derived exosomes enriched on the functionalized SiO_2_‐chip was determined to be 10000 particles mL^−1^, as shown by the red dashed line in Figure 3F,G. Furthermore, the qRT‐PCR analysis of LUCAT‐1 and EGFR‐AS‐1 revealed a single melting curve peak, indicating the presence of a unique product and confirming the specificity of the reaction (Figure 3H,I). Compared to commercially available exosome detection kits (2.9 × 10 000 000 particles mL^−1^), our method demonstrated a lower detection limit.^[^ ^32^ ^]^ We collected HCC patient plasma volumes of 10, 20, 30, 40, 50, 60, 70, and 80 µL^−1^. After inputting these volumes into the chip and performing qRT‐PCR detection, we observed that when the input volume is <40 µL^−1^, the ct value exceeds 35. Typically, in practical testing, a ct value below 30 indicates more stable PCR detection. Therefore, we recommend a plasma input volume of 70 µL^−1^ or more for more reliable detection during testing (Figure S6, Supporting Information). The exceptional sensitivity achieved by our functionalized SiO_2_‐chip at such low volumes can be attributed to the efficient enrichment of exosomes facilitated by the porous structure and rough surface of the scaffold and the wide range of nanopores between the microspheres.

### 2.4 Detection of HCC‐Associated lncRNAs in Exosome Enriched by SiO_2_‐Chip for the Diagnosis of Patients with HCC

**Figure 4:**
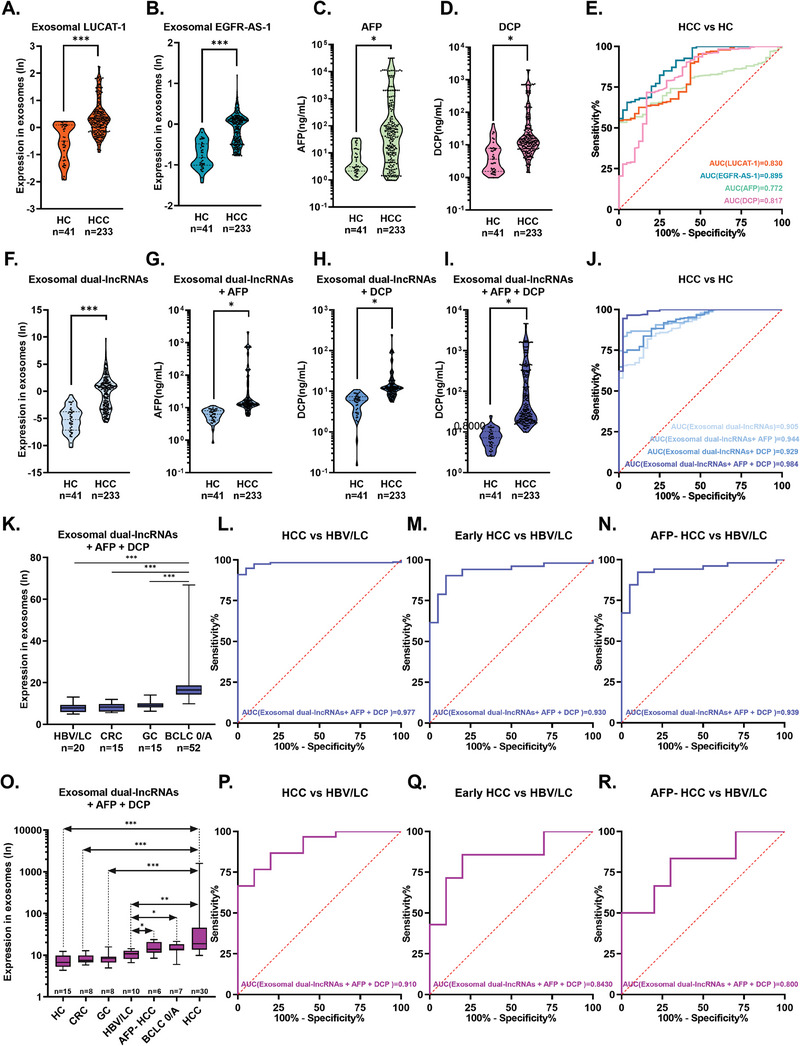
Performance evaluation of the transcriptomic signature in exosomes enriched using SiO_2_‐chip in clinical cohorts by qRT‐PCR. A–D) Levels of exosomal LUCAT‐1 A), exosomal EGFR‐AS‐1 B), serum AFP C), and serum DCP D) in the ZHWU‐1 cohort, presented as violin plots displaying all data points, with significance assessed through t‐test. E) ROC curve analysis for exosomal LUCAT‐1, exosomal EGFR‐AS‐1, serum AFP, and serum DCP in the ZHWU‐1 cohort. F–I) Levels of exosomal dual‐lncRNAs F), exosomal dual‐lncRNAs + serum AFP G), exosomal dual‐lncRNAs + serum DCP H), and exosomal dual‐lncRNAs + serum AFP + serum DCP (I) in the ZHWU‐1 cohort, presented as violin plots displaying all data points, with significance assessed through t‐test. J) ROC curve analysis for different combinations of exosomal LUCAT‐1, exosomal EGFR‐AS‐1, serum AFP, and serum DCP in the ZHWU‐1 cohort. K) Levels of exosomal dual‐lncRNAs + serum AFP + serum DCP in the ZHWU‐1 cohort, presented as the range from minimum to maximum values, with significance assessed using *t*‐test. (L‐N) ROC curve analysis for the level of exosomal dual‐lncRNAs + serum AFP + serum DCP in the ZHWU‐1 cohort. O) Levels of exosomal dual‐lncRNAs + serum AFP + serum DCP in the RHWU cohort, presented as the range from minimum to maximum values, with significance assessed using t‐test. P–R) ROC curve analysis for the level of exosomal dual‐lncRNAs + serum AFP + serum DCP in the RHWU cohort. Abbreviations: HC (healthy control), HBV/LC (patients with hepatitis caused by hepatitis B virus infection and patients with liver cirrhosis) HCC, (hepatocellular carcinoma;), AFP‐ HCC (HCC patients with negative alpha‐fetoprotein), CRC (colorectal cancer patients) GC, (gastric cancer patients), and BCLC 0/A (patients with Barcelona Clinic Liver Cancer stage 0/A, which corresponds to early‐stage HCC). [Please note that the sensitivity and specificity of all markers in each cohort are presented in Table S5, Supporting Information.]

Plasma samples were collected from patients in the Zhongnan Hospital of Wuhan University‐1 Cohort (ZHWU‐1 Cohort). Subsequently, we performed exosome enrichment using a SiO_2_‐chip and conducted qRT‐PCR analysis. Both LUCAT‐1 and EGFR‐AS‐1 were detected, demonstrating the robustness of the earlier biomarker discovery studies. First, we analyzed individual levels of exosomal LUCAT‐1, exosomal EGFR‐AS‐1, serum AFP, and serum DCP in the ZHWU‐1 Cohort. The results showed significant differences in these four markers between healthy individuals and patients with HCC (**Figure** **4**A,D). The diagnostic performances of exosomal LUCAT‐1 and EGFR‐AS‐1 were higher than those of serum AFP and DCP (Figure 4E).

To explore the optimal diagnostic model, we combined exosomal LUCAT‐1, exosomal EGFR‐AS‐1, serum AFP, and serum DCP in different combinations and compared their diagnostic performance. We found that the combination of these markers had better diagnostic performance than the individual markers, with the best performance achieved by the model combining all four markers (Figure 4F–J). The formula for the combination model is as follows:

(1) $$\begin{array}{ccc} \mathrm{COMBINATION}\_1 & = & \left(\mathrm{exosomal}\;\mathrm{LUCAT}1\times 0.062\right)\\ & & +\left(\mathrm{exosomal}\;\mathrm{EGFR}-\mathrm{AS}-1\times 0.355\right) \end{array}$$

(2) $$\begin{array}{ccc} \mathrm{COMBINATION}\_2 & = & \left(\mathrm{exosomal}\;\mathrm{LUCAT}1\times 0.058\right)\\ & & +\left(\mathrm{exosomal}\;\mathrm{EGFR}-\mathrm{AS}-1\times 0.356\right)\\ & & +\left(\mathrm{serum}\;\mathrm{AFP}\times 0.000005\right) \end{array}$$

(3) $$\begin{array}{ccc} \mathrm{COMBINATION}\_3 & = & \left(\mathrm{exosomal}\;\mathrm{LUCAT}1\times 0.054\right)\\ & & +\left(\mathrm{exosomal}\;\mathrm{EGFR}-\mathrm{AS}-1\times 0.367\right)\\ & & +\left(\mathrm{serum}\;\mathrm{DCP}\times 0.000173\right) \end{array}$$

(4) $$\begin{array}{ccc} \mathrm{COMBINATION}\_4 & = & \left(\mathrm{exosomal}\;\mathrm{LUCAT}1\times 0.05\right)\\ & & +\left(\mathrm{exosomal}\;\mathrm{EGFR}-\mathrm{AS}-1\times 0.368\right)\\ & & +\left(\mathrm{serum}\;\mathrm{AFP}\times 0.000004471\right)\\ & & +\left(\mathrm{serum}\;\mathrm{DCP}\times 0.000141\right) \end{array}$$

Furthermore, we observed that when exosomal LUCAT‐1, exosomal EGFR‐AS‐1, serum AFP, and serum DCP were combined, it not only distinguished early stage HCC patients from patients with other digestive system tumors but also differentiated early stage HCC patients from patients with precancerous lesions (hepatitis B infection or liver cirrhosis). This indicates that a combination of exosomal transcriptomic signatures and clinical markers can be used for both diagnostic and early detection (Figure 4K–N). The combination of exosomal dual‐lncRNAs with serum AFP and serum DCP not only effectively distinguished HCC from HC but also showed great potential for early HCC diagnosis, with statistically significant differences in the area under curve (AUC), thereby complementing the existing clinical markers [pairwise comparison of receiver operating characteristic curve (ROC) curves of each marker is shown in Table S4, Supporting Information].

Next, in the independent validation cohort, named the Renmin Hospital of Wuhan University Cohort (RHWU Cohort), we studied the diagnostic potential of exosomal transcriptomic signature detection in combination with clinical HCC markers using the same COMBINATION_4 model parameters as in the training cohort. Based on the data obtained from the RHWU Cohort, the combination of exosomal LUCAT‐1, exosomal EGFR‐AS‐1, serum AFP, and serum DCP effectively differentiated healthy individuals from patients with HCC (Figure 4O) and displayed superior characteristics (Figure S7 and Table S4, Supporting Information). Similar to the training cohort, the combination of an exosomal transcriptomic signature with AFP and DCP allowed for diagnostic and early detection (Figure 4P–R).

### 2.5 Use of Exosomal HCC‐associated lncRNAs Levels in Monitoring the Progression of HCC

**Figure 5:**
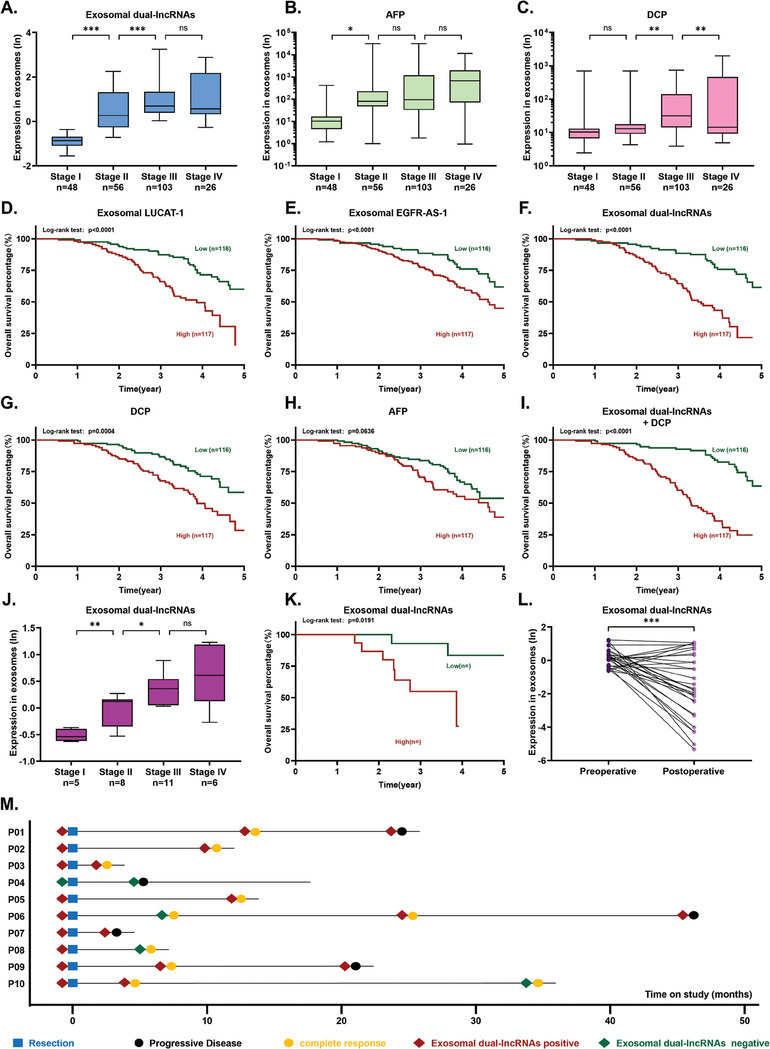
The exosomal shows potential as a biomarker for early recurrence prediction. A–C) Scatter plots display the expression levels of the exosomal dual‐lncRNAs, AFP, and DCP of HCC patients with different clinical stage in the ZHWU‐1 cohort, presented as the range from minimum to maximum values, with significance assessed using *t*‐test. D–I) Kaplan–Meier curves of OS based on different biomarker in the ZHWU‐1 cohort. *p*‐Values were calculated using the log‐rank test. J) Scatter plots display the expression levels of the Exosomal dual‐lncRNAs with different clinical stage in the RHWU cohort, presented as the range from minimum to maximum values, with significance assessed using *t*‐test. K) Kaplan–Meier curves of OS based on exosomal dual‐lncRNAs in the RHWU cohort. *p*‐Value was calculated using the log‐rank test. L) Exosomal dual‐lncRNAs level of 30 paired plasm samples which were collected before and 5 days after liver cancer resection, originating from HCC patients in the RHWU cohort. *p*‐Value was calculated using paired *t* test. M) Swimmer plots illustrating exosomal dual‐lncRNAs levels at various time points in the ZHWU‐2 Cohort. Abbreviations: OS (overall survival).

To determine whether the levels of Exosomal HCC‐associated lncRNAs were associated with HCC progression, we collected clinical information from patients in the ZHWU‐1 cohort. We found that the levels of exosomal dual lncRNAs gradually increased from clinical stage I to IV (Stage I vs II, *p* < 0.001; Stage II vs III, *p* < 0.001) (**Figure** **5**A). In contrast, serum AFP levels showed statistical differences only between stages I and II (Figure 5B), while serum DCP levels increased progressively from clinical stages I to IV (Stage II vs III, *p* < 0.001; Stage III vs IV, *p* < 0.001) (Figure 5C).

To further evaluate the potential value of exosomal dual lncRNAs in HCC prognosis, we divided patients into high and low groups based on the median levels of different biomarkers in the ZHWU‐1 cohort. Kaplan‐Meier analysis revealed that patients with HCC with high exosomal LUCAT‐1 levels had a significantly shorter overall survival (OS) compared to those with low exosomal LUCAT‐1 levels (Figure 5D, HR 2.920 [95% confidence interval (CI) 1.860 to 4.584]). Similar trends were observed for exosomal EGFR‐AS‐1 (Figure 5E, HR 1.827 [95% CI 1.247–2.677]), exosomal dual lncRNAs (Figure 5F; HR 4.043 [95% CI 2.559–6.389]), and serum DCP (Figure 5G; HR 2.199 [95% CI 1.423–3.399]). However, no statistically significant differences in OS were observed between patients with high and low groups based on serum AFP levels. Notably, the combination of exosomal dual‐lncRNAs and serum DCP levels improved the prediction of OS (Figure 5I, HR 5.426 [95% CI 3.427–8.591]).

We further evaluated the prognostic predictive ability of exosomal dual lncRNAs in patients with HCC in the RHWU Cohort. Similar to the ZHWU‐1 cohort, exosomal dual‐lncRNA levels gradually increased from clinical stages I to IV (Stage I vs II, *p* < 0.001; Stage II vs III, *p* < 0.001) (Figure 5J). Kaplan‐Meier analysis revealed that patients with HCC with high exosomal dual‐lncRNA levels had a significantly shorter OS than those with low exosomal dual‐lncRNA levels (Figure 5K, HR 5.068 [95% CI 1.304–19.70]).

Subsequently, we analyzed 30 paired plasma samples collected before and five days after liver cancer resection from patients with HCC in the RHWU Cohort. Among them, 22 patients showed significantly decreased exosomal dual‐lncRNA levels after surgery (*p* < 0.001) (Figure 5L).

Swimmer plots were used to depict exosomal dual‐lncRNA levels at different time points in the Zhongnan Hospital of Wuhan University‐2 Cohort (ZHWU‐2 Cohort) (Figure 5M). Among the five patients with disease progression (PD) or recurrence, three were exosomal dual‐lncRNA‐positive in the training group, while only one out of the three exosomal dual‐lncRNA‐negative patients experienced recurrence or progressive disease. Notably, patient P06, who was exosomal dual lncRNA‐positive, showed recurrence before any positive findings on imaging, indicating that exosomal dual lncRNAs could serve as an early warning for HCC recurrence.

Together, these results indicate that exosomal dual‐lncRNA levels significantly increase with HCC progression from the early to advanced stages and can serve as prognostic biomarkers for HCC.

## 3 Discussion

Owing to their unique advantages, exosomes offer remarkable promise as cancer biomarkers. Encased within lipid bilayers, exosomes are shielded from enzymatic degradation and external influences, thus ensuring cargo stability, facilitating long‐term storage, and efficient transport. These vesicles carry specific biomolecules, including proteins, nucleic acids (RNA and DNA), and lipids, which are characteristic of their cells of origin, enabling highly sensitive and specific detection of cancer‐related molecular signatures. Continuous release by tumor cells allows real‐time disease monitoring, making exosomes ideal for dynamic disease management. Moreover, their noninvasive isolation makes exosomes excellent candidates for liquid biopsies, enabling early cancer detection, disease progression monitoring, and treatment response evaluation. However, current exosome isolation techniques such as ultracentrifugation and emerging sorting methods^[^ ^23^, ^33^ ^]^ require further advancement to achieve rapid and efficient enrichment.

Our study demonstrated the multifaceted advantages of the SiO_2_‐chip method. First, the continuous porous structure of the chip facilitates fluid flow from laminar to turbulent flow, significantly enhancing the collision frequency between exosomes and the substrates. Second, by co‐assembling the silicon dioxide microspheres of different sizes, the pore size of the scaffold was effectively reduced and the surface area was increased for exosome capture. Nanoscale pores between the microspheres minimized the boundary effects, further enhancing exosome binding to the scaffold. These cumulative effects synergistically improved the exosome capture efficiency of the chip. Our SiO_2_‐chip successfully isolates exosomes from plasma samples, achieving a remarkable detection limit of as low as 10000 particles mL^−1^. Remarkably, the entire analysis requires only 40 µL of plasma and can be completed within a rapid 10‐min timeframe.

Considering the advantages of SiO_2_‐chip enrichment, we have made significant strides in the discovery of HCC‐related exosomal biomarkers. The identified lncRNAs were enriched in crucial pathways, such as the regulation of cell migration, EGF/EGFR signaling, programmed cell death, PIP3 activation of AKT signaling, and regulation of the Wnt pathway, all of which are critical for HCC progression.

Furthermore, our method addresses the challenges associated with stable exosome acquisition and specialized separation in clinical settings. Distinguishing HCC from other digestive tract tumors and detecting high‐risk versus early stage HCC remains challenging. However, the exosomal lncRNA biomarkers identified using our enrichment chip not only distinguished liver cancer from other gastrointestinal tumors, but also distinguished high‐risk lesions from early stage HCC. Given that a significant number of patients with HCC transition from HBV infection to cirrhosis, and then to HCC,^[^ ^34^ ^]^ screening individuals with precancerous lesions is imperative. Importantly, combining our exosomal lncRNA markers with existing clinical markers, such as AFP and DCP, significantly improved diagnostic efficacy. Additionally, our exosomal lncRNA markers show promise for non‐invasive HCC prognosis prediction and disease monitoring, outperforming imaging and circulating biomarkers in early relapse detection when combined with DCP.

The versatility of our exosomal lncRNA markers allows them to be combined with various serum markers for personalized and precise HCC screening, thereby transforming the current “one‐size‐fits‐all” approach. This has the potential to significantly improve the early detection and prognosis of HCC. In conclusion, our exosomal lncRNA markers based on the innovative SiO_2_‐chip enrichment method hold great promise for revolutionizing HCC screening and management strategies, ultimately leading to improved early detection and prognostic prediction of HCC.

Nevertheless, this study has several potential limitations. First, although we conducted functional analyses of lncRNAs at the molecular data science level, further biological experiments are necessary to fully understand their role in HCC. Second, to better suit clinical applications, integrating exosome isolation and detection into a single technology with a higher throughput would be advantageous. Finally, the relatively small sample size in our study, albeit encompassing multiple ethnicities and races, warrants larger prospective studies from diverse sources to successfully translate these findings to routine clinical settings.

## 4 Conclusion

In conclusion, our study demonstrated the remarkable effectiveness of the SiO_2_‐chip for exosome enrichment. The comprehensive and systematic discovery of biomarkers, coupled with successful clinical validation, provides compelling evidence for the clinical significance of noninvasive liquid biopsy detection based on SiO_2_‐chip‐enriched exosomal transcriptomic profiles for the early detection and prognostic monitoring of patients with HCC.

## 5 Experimental Section

### Preparation of 3D Porous Scaffold

The nickel (Ni) foam was cut into 33 mm× 8 mm × 1 mm. They were then subjected to ultrasonic cleaning in ethanol and ultrapure water for 30 min. Polydimethylsiloxane (PDMS) prepolymer and cross‐linker were thoroughly mixed in the ratio of 10:1 and put in −20 °C refrigerator overnight to defoam. Two pieces of foamed nickel were inserted into the PDMS mixture, which was subsequently centrifuged at 8000 rpm for 5 min to ensure complete coverage of the PDMS on the foamed nickel. The PDMS‐coated nickel foam was transferred to a new centrifuge tube and centrifuged again at 4250 rpm for 4 min to remove excess PDMS, resulting in a PDMS layer covering the nickel foam surface. The nickel foam coated with PDMS was then heated at 75 °C for 3 h to make PDMS solidification. This process was repeated to apply a second layer of PDMS to the Ni foam skeleton. After curing, the PDMS‐coated Ni foam was immersed in nitric acid and reacted in a shaking bed for 5 h for etching. It was then washed three times with water and dried at 75°C, yielding a 3D porous PDMS scaffold with self‐supporting properties.

The PDMS scaffold was treated with oxygen plasma for 5 min, followed by immediate immersion in a 1 mg mL^−1^ solution of dopamine (DA)/Tris‐HCl. The material was allowed to react on a shaking bed for 2 h and then left to rest for 48 h. The excess pDA on the material surface was washed away with ultrapure water, resulting in a pDA/PDMS scaffold with a negatively charged surface. The pDA/PDMS scaffold was immersed in a solution of 1% positively charged polyethyleneimine (PEI) and shaken for 3 h to obtain a PEI/pDA/PDMS scaffold. After washing with ultrapure water three times, the scaffold was immersed in a solution of carboxylated SiO_2_ microspheres and shaken for 6 h to obtain SiO_2_ microsphere‐coated PDMS scaffold. This operation was repeated to assemble three layers of SiO_2_ microspheres on the PDMS scaffold, resulting in the generation of a 3D porous PDMS scaffold coated with SiO_2_ microspheres (denoted as SiO_2_/PDMS scaffold).

### Functionalization of 3D SiO_2_/PDMS Scaffold

First, the 3D SiO_2_/PDMS scaffold was treated with oxygen plasma for 5 min, incubated with 5% sodium carboxyethylsilanetriol solution on a shaker for 4 h, and subsequently washed with PBS three times. The scaffold was then immersed in a solution of 10 mM 1‐(3‐dimethylaminopropyl)−3‐ethylcarbodiimide hydrochloride (EDC) and 20 mM N‐hydroxy succinimide (NHS) for 1 h to activate the carboxyl groups on the scaffold. Thirdly, after washing by PBS for 3 times, 50 µg mL^‐1^ streptavidin (SA) was used to incubate with the scaffold for 4 h. The scaffolds were then washed with PBS. Finally, the scaffold was immersed into the solution of 10 µg mL^‐1^ biotin‐labeled anti‐CD63 antibody for 1 h. It was then washed by PBS and stored at 4 °C for future use. DyLight 594‐labeled goat anti‐mouse IgG was incubated with the scaffold for 1 h to identify successful conjugation of the antibody on the scaffold, while the scaffold without conjugation of the antibody was used as a control. After washing with PBS, these scaffolds were observed using fluorescence microscopy and laser scanning confocal microscopy.

### Cell Culture and Exosome Extraction

The cell lines were cultured in 1640 medium supplemented with 10% fetal bovine serum and placed in a 37 °C incubator with 5% carbon dioxide. Once the cell density reached 80%, the supernatant was removed, and serum‐free RPMI 1640 medium was added. Following a 36‐h starvation treatment, cell culture supernatants were collected. The ultracentrifugation procedure described previously was used to extract exosomes from cell supernatants.^[^ ^35^ ^]^ The supernatant was used as the sample, and the cells were separated by centrifugation at a low speed of 300 × *g* for 10 min. The supernatant was further centrifuged at 2000 × *g* for 10 min to eliminate dead cells. The remaining supernatant was centrifuged for 30 min at 10000 × *g* to remove the cell debris. Subsequently, the resulting supernatant was centrifuged for 70 min at 100, 000 × *g*, resulting in coarse exudate precipitation. The exudate was precipitated with PBS and subjected to another round of centrifugation for 70 min at 100000 × *g*. Finally, the obtained sediment was suspended in PBS to yield pure exosomes.

### Nanoparticle Tracking Analysis

The size and concentration of exosomes were determined using nanoparticle tracking analysis (NTA) with a Zetaview‐PMX120‐Z instrument and Zeta View software (version 8.05.14SP7).

### Isolation of Exosomes Using the SiO_2_‐Chip

In this study, a customized microchip with a single‐channel manifold (geometric size:25 mm × 4 mm ×0.6 mm, length × width × thickness) was used. The antibody‐functionalized 3D SiO_2_/PDMS scaffold was cut to an appropriate size and embedded in a customized single‐channel manifold to construct the SiO_2_‐chip. Prior to using the chip for exosome capture, a 5% BSA solution was used to block the syringe, hose, and chip for 1 h. This step helped reduce the nonspecific adsorption of exosomes. After washing syringe, hose and chip with PBS, the samples containing exosomes were pumped into the SiO_2_‐chip at a flow rate of 10 µL min^‐1^, followed by PBS washing at a flow rate of 10 µL min^‐1^ for 5 min. Next, the exosomes were captured on the chip.

### Western Blot Analysis

RIPPA was mixed with PMSF at a ratio of 100:1, and then 30 µL of the mixture was introduced into the chip to extract the enriched exosomal proteins. A Sodium Dodecyl Sulfate Polyacrylamide Gel Electrophoresis (SDS‐PAGE) preparation kit was used to separate proteins. The proteins were subsequently transferred onto polyvinylidene fluoride (PVDF) membranes, which were blocked at room temperature for 1 h. The membranes were then incubated overnight at 4 °C with primary antibody dilutions. The antibodies used for western blot analysis were anti‐AFP (Abbott), Anti‐CK19 (Abbott), anti‐ALIX (Abbott), and Anti‐CD9 (Abbott). The membranes were then rinsed three times with TBST for 10 min each with TBST solution. The membranes were then immersed in a diluent containing secondary antibodies for 60 min at room temperature. Enhanced chemiluminescence was used for image capture. All antibodies were diluted to a ratio of 1:2000.

### Quantitative Real‐time Polymerase Chain Reaction (qRT‐PCR)

After injecting the lysate into the exosome‐enriched chip, total RNA was extracted using the TRIzol reagent. The extracted RNA was then subjected to reverse transcription using ReverTra Ace qPCR RT Master Mix (Code No. FSQ‐201) to generate cDNA complementary to the single‐stranded RNA. Real‐time quantification of cDNA was performed using a SYBR Green PCR kit and cycle threshold (Ct) values were recorded.The primer sequences used for qPCR detection are listed in **Table** **1**. The relative expression of each gene was determined by calculating the mean value and applying the 2^−∆∆CT^ method to assess statistical significance between groups. Duplicate wells were used, and the mean values obtained from the wells were considered the final experimental results. All procedures were performed according to manufacturer's instructions.

**Table 1:** The primer sequence of genes.

Gene	Primer sequence
GAPDH	Forward: 5′‐GGTCTCCTCTGACTTCAACA‐3′ Reverse: 5′‐GTGAGGGTCTCTCTCTTCCT‐3′
LUCAT1	Forward: 5′‐CTCAGACAATGCCCAGACCTC‐3′ Reverse: 5′‐GCCAGGACGGAGATCAGATG‐3′
EGFR‐AS1	Forward: 5′‐CCTTCCAGGTGAAGACGCAT‐3′Reverse: 5′‐AAACGTCCCTGTGCTAGGTC‐3′

### Drawing Regulatory Networks

The target genes of the lncRNAs were predicted using LncTarD 2.0 (http://www.bio‐database.com/). Screened target genes are listed in Table S2 (Supporting Information). Enrichment analysis of screened target genes was performed using the g:Profiler tool (http://biit.cs.ut.ee/gprofiler/gost). Background databases included GO (Gene Ontology), KEGG (Kyoto Encyclopedia of Genes and Genomes), Reactome, and Wiki Pathways. *p*‐value correction was performed using the g: SCS method, and entries with corrected *p* < 0.05 were considered significantly enriched, as indicated in Table S3 (Supporting Information). Finally, regulatory networks were constructed based on the results using Microsoft PowerPoint.

### RNA Sequencing

To prepare libraries for total RNA sequencing, the SMARTer Stranded Total RNA Seq Kit v2 Pico Input Mammalian (Clontech, 634413) was employed. The size of each library was determined using an Agilent High Sensitivity DNA Kit (Agilent, 5067‐4626), and the concentration was measured using a Library Quantification Kit (Clontech, 638324). Alternatively, library quantification was performed using the Qubit dsDNA HS Assay Kit (Thermo Fisher Scientific, Q32854).

Subsequently, the libraries were pooled in equimolar amounts, and their sizes and concentrations were measured. Library sequencing was performed using the HiSeq 2500 platform (Illumina).

### Study Population

**Figure 6:**
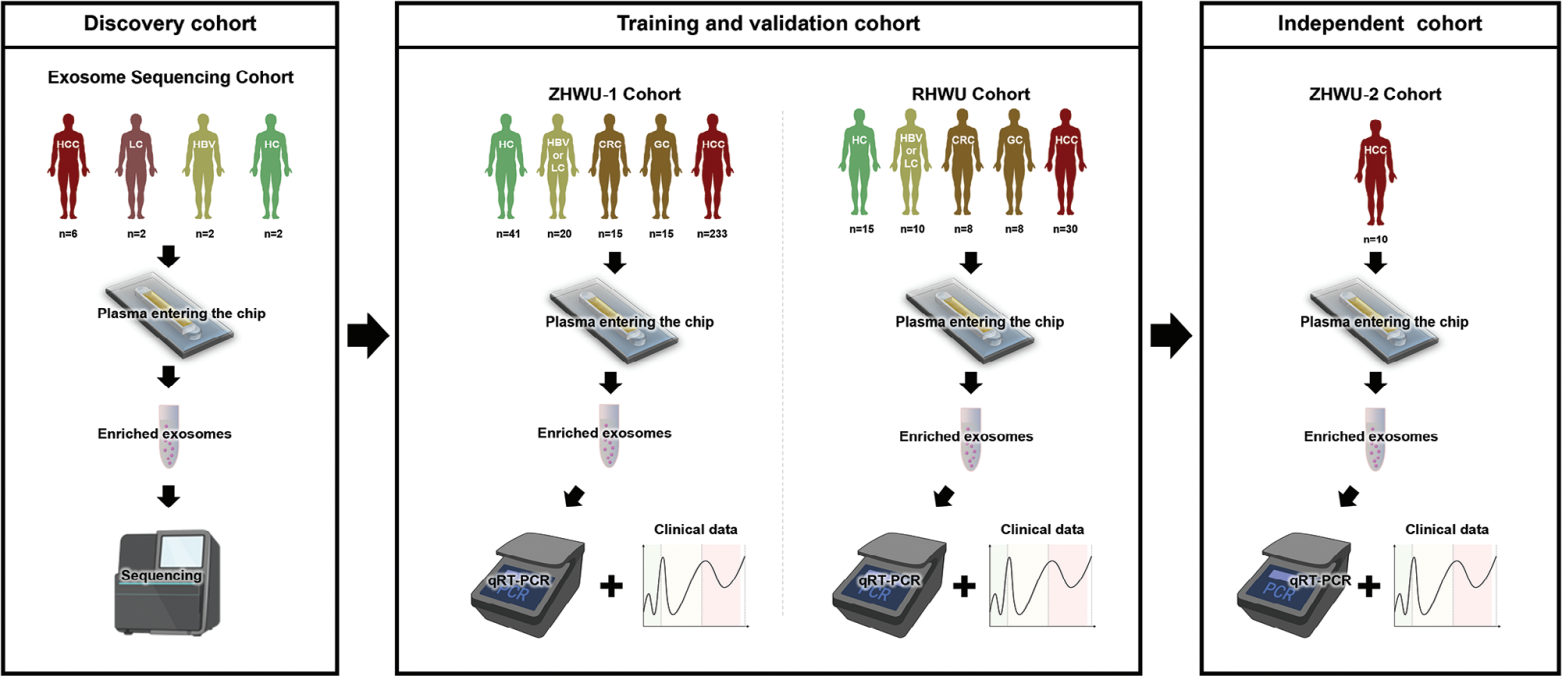
Illustration of the clinical samples in this study.

The study population consisted of four distinct cohorts as depicted in **Figure** **6**: discovery, training, validation, and independence. These cohorts were sourced from two institutions: Zhongnan Hospital of Wuhan University (ZHWU) with three subsets: ES, ZHWU‐1, and ZHWU‐2, and Renmin Hospital of Wuhan University (RHWU). The ZHWU‐1 cohort was used for model development, and the ZHWU‐2 and RHWU cohorts were used for model validation. Individuals in the healthy control group were excluded if they had a history of malignancy within the past 6 months; severe oral diseases; diabetes; pulmonary, renal, or hepatic impairments; significant immune alterations; or a history of cardiovascular events. Detailed information on each cohort is provided in **Table** **2**. To ensure ethical compliance, the study protocol was approved by the Ethics Committee of Zhongnan Hospital of Wuhan University and Renmin Hospital of Wuhan University, adhering to the principles outlined in the Declaration of Helsinki. The approval number for the use of human samples were 2 022 003.

**Table 2:** Characteristics of study participants.

Exosome sequencing cohort	HCC (*n* = 6)	Control (*n* = 6)
Age, yr	64(48‐82)	66 (52‐78)
Sex		
Male	5(83.33)	4(66.67)
Female	1(16.67)	2 (33.33)
TNM stage		
I	1(16.67)	NA
II	2 (33.33)	
III	2 (33.33)	
IV	1(16.67)	

ZHWU‐1 Cohort	HCC (*n* = 233)	Control (*n* = 91)
Age, yr.	67(47‐77)	61 (50‐73)
Sex		
Male	202(86.70)	67(73.63)
Female	31(13.30)	24(26.37)
TNM stage		
I	48(20.60)	NA
II	56(24.03)	
III	103(44.21)	
IV	26(11.16)	

RHWU Cohort	HCC (*n* = 30)	Control (*n* = 46)
Age, yr	68(61‐75)	64(59‐78)
Sex		
Male	26(86.67)	38(82.61)
Female	4(13.33)	8(17.39)
TNM stage		
I	5(16.67)	NA
II	8 (26.67)	
III	11(36.67)	
IV	6(20.00)	

ZHWU‐2 Cohort	HCC (*n* = 10)	
Age, yr.	71(66‐82)	
Sex		
Male	10(100)	
Female	0(0)	

### Statistical Analysis

Graphical representation and statistical analysis were conducted using Prism 8.0 software. The number of experimental replicates is specified in the Figure legends. To assess significance, significance analysis, constructed ROC curves, and calculated the area under the curve (AUC) were performed using GraphPad Prism 8. The 95% confidence intervals (CIs) were calculated using the binomial distribution. To develop a comprehensive diagnostic model utilizing different biomarkers, multivariate logistic regression was employed. This allowed to assess the contribution of each biomarker to the model, considering both individual effects and potential interactions. To determine the optimal threshold for distinguishing the presence and absence of the disease, receiver operating characteristic (ROC) curves were utilized. The best threshold was determined based on Youden's index, which maximizes both sensitivity and specificity. Additionally, Kaplan‐Meier analysis to calculate the overall survival curve was employed. To assess all significant differences, a two‐tailed test was used, and a *p*‐value <0.05 was considered statistically significant, indicating the presence of a significant difference. In figures, *p* values are indicated by asterisks. * *p* < 0.05, ** *p* < 0.01, *** *p* < 0.001, ns *p*>0.05.

## Conflict of Interest

The authors declare no conflict of interest.

## Supporting information

Supporting Information

Supporting Table 1

Supporting Table 2

Supporting Table 3

Supporting Table 4

Supporting Table 5

## Data Availability

The data that support the findings of this study are available on request from the corresponding author. The data are not publicly available due to privacy or ethical restrictions.
