# Supplementary material for: Transcriptomic Signature of 3D Hierarchical Porous Chip Enriched Exosomes for Early Detection and Progression Monitoring of Hepatocellular Carcinoma
Source: Adv Sci (Weinh). 2024 Feb 7;11(14):2305204. doi: 10.1002/advs.202305204 (PMC11005692; doi:10.1002/advs.202305204)
Supplement: Supplementary file 1 — Supporting Information [file ADVS-11-2305204-s004.pdf]

ADVANCED SCIENCE
Open Access

# Supporting Information

for *Adv. Sci.*, DOI 10.1002/advs.202305204

Transcriptomic Signature of 3D Hierarchical Porous Chip Enriched Exosomes for Early Detection and Progression Monitoring of Hepatocellular Carcinoma

*Kezhen Yi, Yike Wang, Yuan Rong, Yiru Bao, Yingxue Liang, Yiyi Chen, Fusheng Liu, Shikun Zhang, Yuan He, Weihuang Liu, Chengliang Zhu, Long Wu, Jin Peng, Hao Chen, Weihua Huang, Yufeng Yuan*, Min Xie* and Fubing Wang**

**Supplemental Figures**

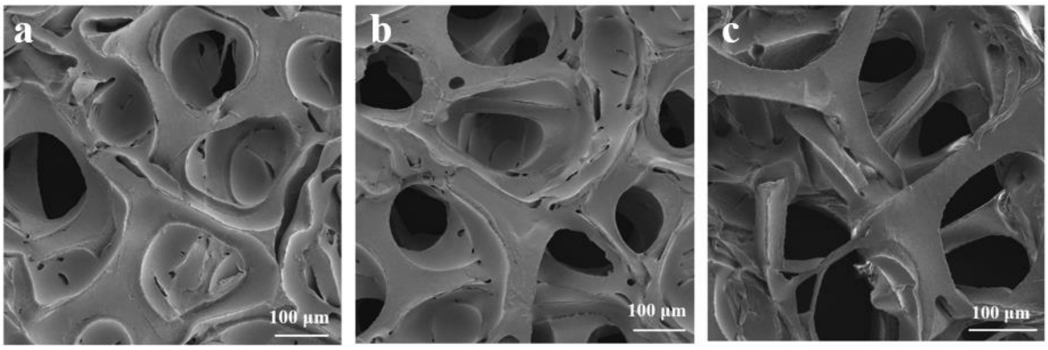


Fig.S1

Optimizing conditions for preparation of 3D porous PDMS scaffold. (a-c) SEM images of 3D porous PDMS scaffold prepared at centrifugal speed of 3750, 4250, 5000 rpm, respectively.

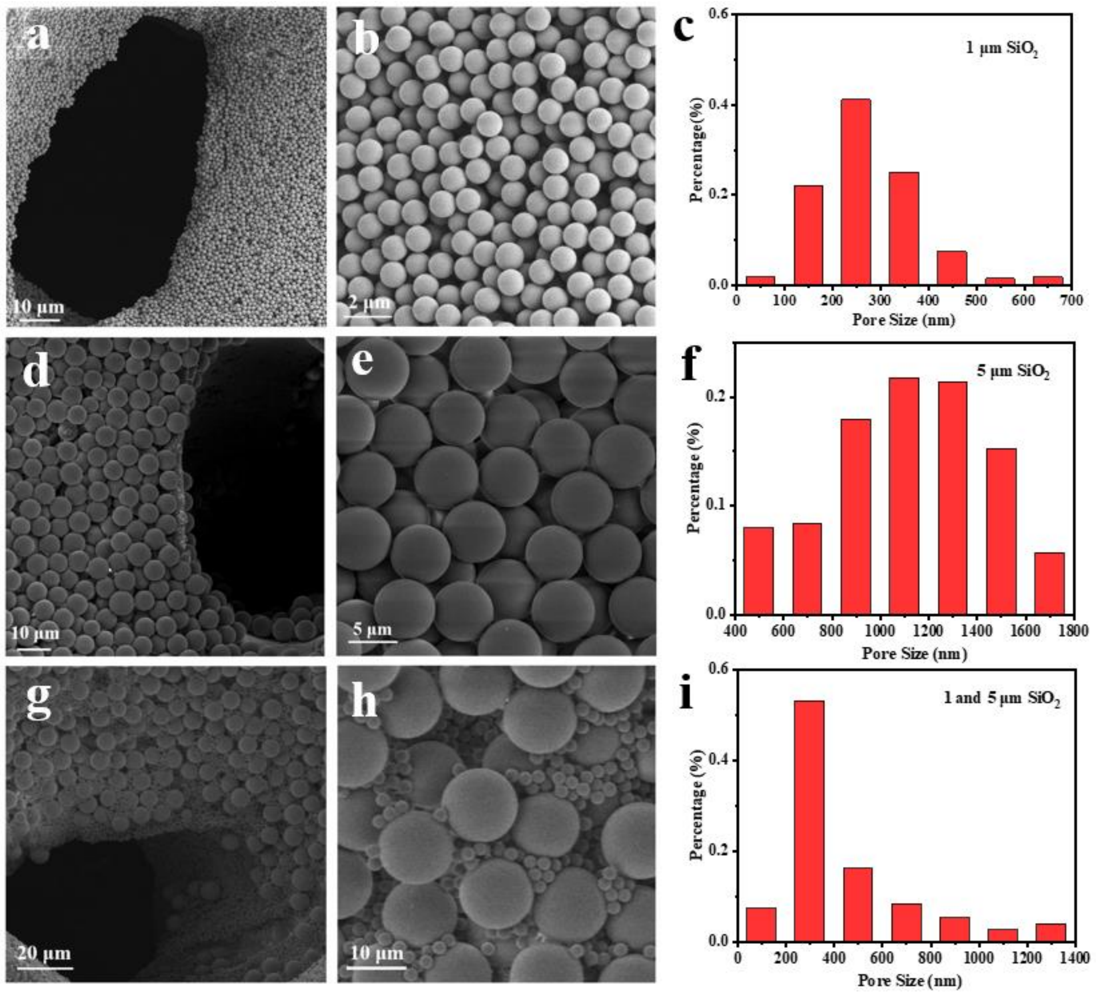


Fig.S2

(a, b, d, e, g, h) SEM images of the 3D PDMS scaffold assembled with 3 layers 1 μm $SiO_2$ (a, b), 5 μm $SiO_2$ (d, e) and co-assembled with 1 and 5μm $SiO_2$ (g, h) at a 1:1 volume ratio. (c, f, i) Pore size distributions between 1 μm $SiO_2$ (c), 5 μm $SiO_2$ (f), 1 and 5μm $SiO_2$ (i).

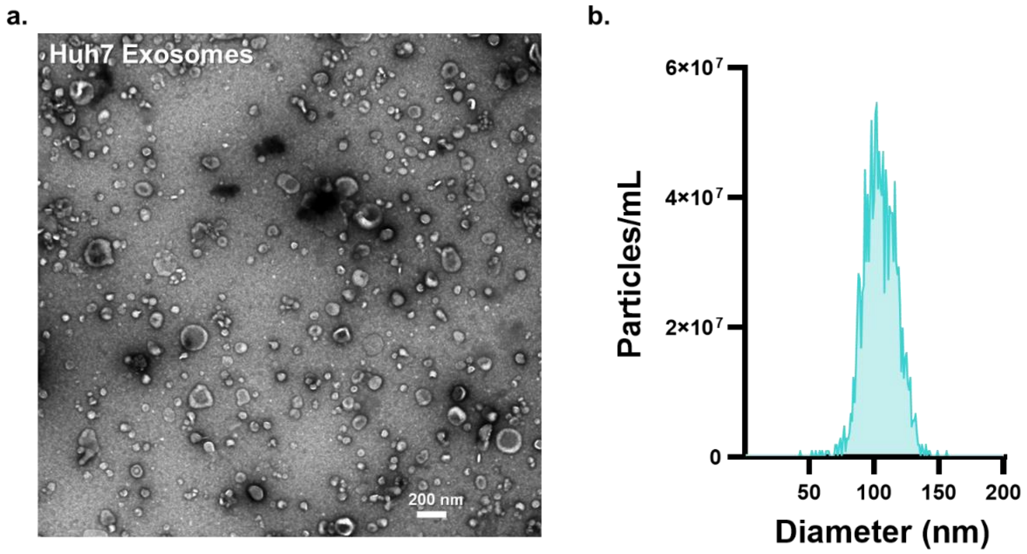


Fig.S3

TEM (a) and NTA(b) results of exosomes derived from Huh7 cell.

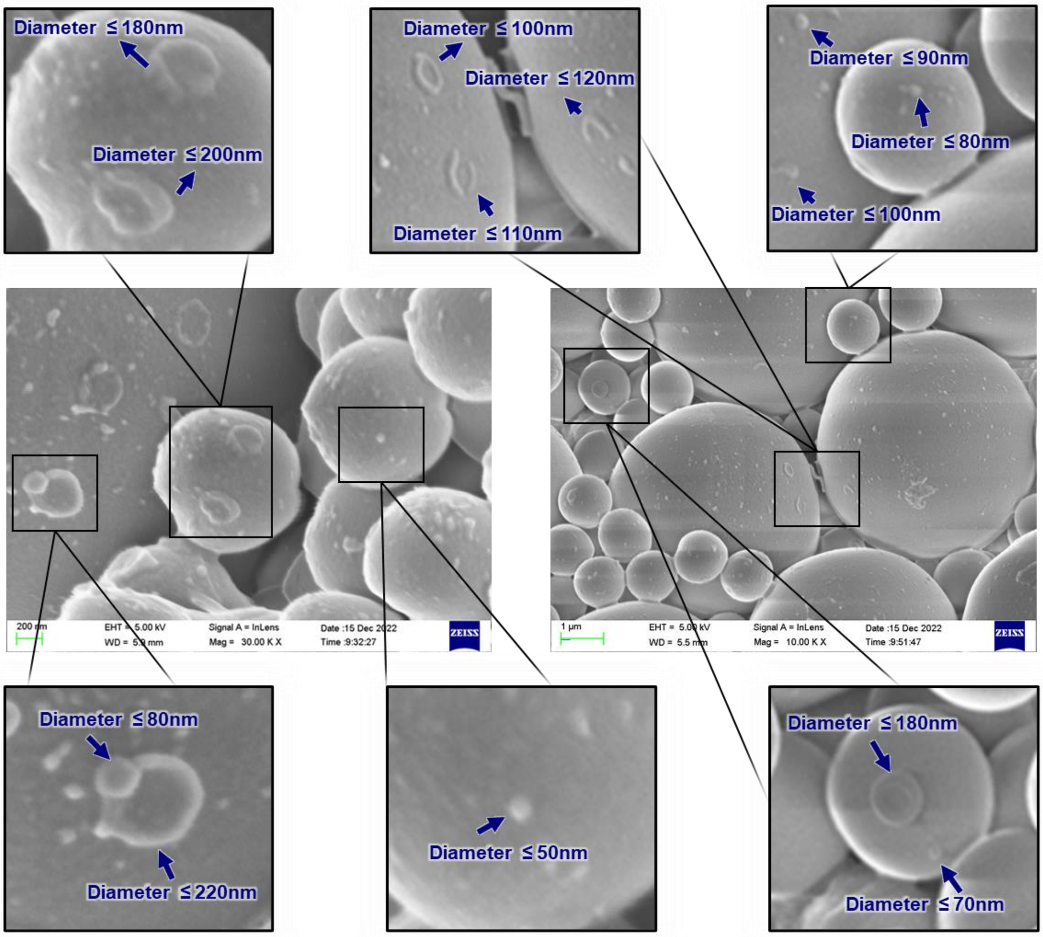


Fig.S4

Based on SEM imaging results after the capture of exosomes on the chip, it is clear that the captured exosomes exhibit a broad size distribution ranging from 50-220nm, with an average size around 100nm.

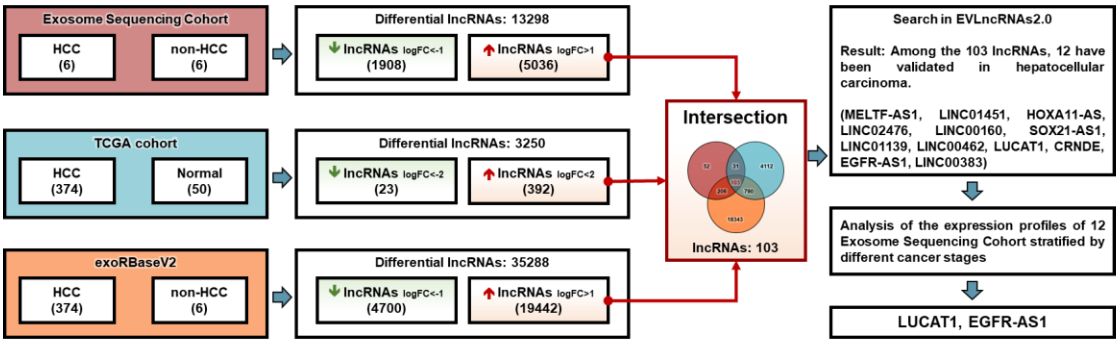


Fig.S5

The datasets we utilized during the selection phase encompass the Exosome Sequencing Cohort, the TCGA cohort, exoRBaseV2, and EVLncRNAs2.0. The Exosome Sequencing Cohort comprises plasma exosome data that we collected ourselves. The TCGA cohort is sourced from a public database and consists of expression data from HCC tissues and normal liver tissues. While this dataset does not include exosomal lncRNA expression profiles, we used it to further ensure that the selected specific exosomal lncRNAs exhibit a strong correlation with HCC. ExoRBaseV2 is a repository derived from RNA-Seq data analysis of human blood exosomes, encompassing circular RNAs (circRNA), long non-coding RNAs (lncRNA), and messenger RNA (mRNA). This dataset was employed to confirm that the selected specific exosomal lncRNAs can be detected in human blood exosomes. EVLncRNAs2.0 is a comprehensive database of functionally verified lncRNAs, validated through low-throughput experiments such as qRT-PCR, knockdown experiments, Northern blotting, and luciferase reporter gene assays. This database was utilized to ensure that the selected specific exosomal lncRNAs can be detected through qRT-PCR.

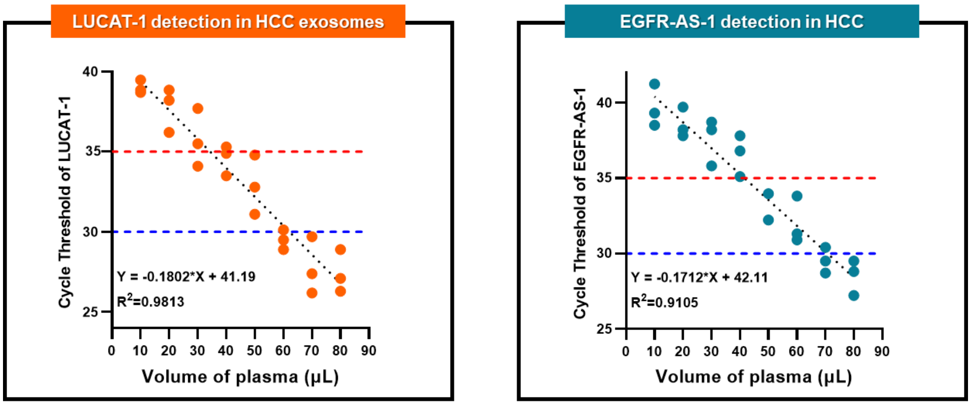


Fig.S6

Sensitivity and linear range of LUCAT-1 and EGFR-AS-1 detecting in exosomes enriched by 3D porous chip device from different volume of HCC plasma (n = 3 independent technical replicates). $R^2$, coefficient of determination.

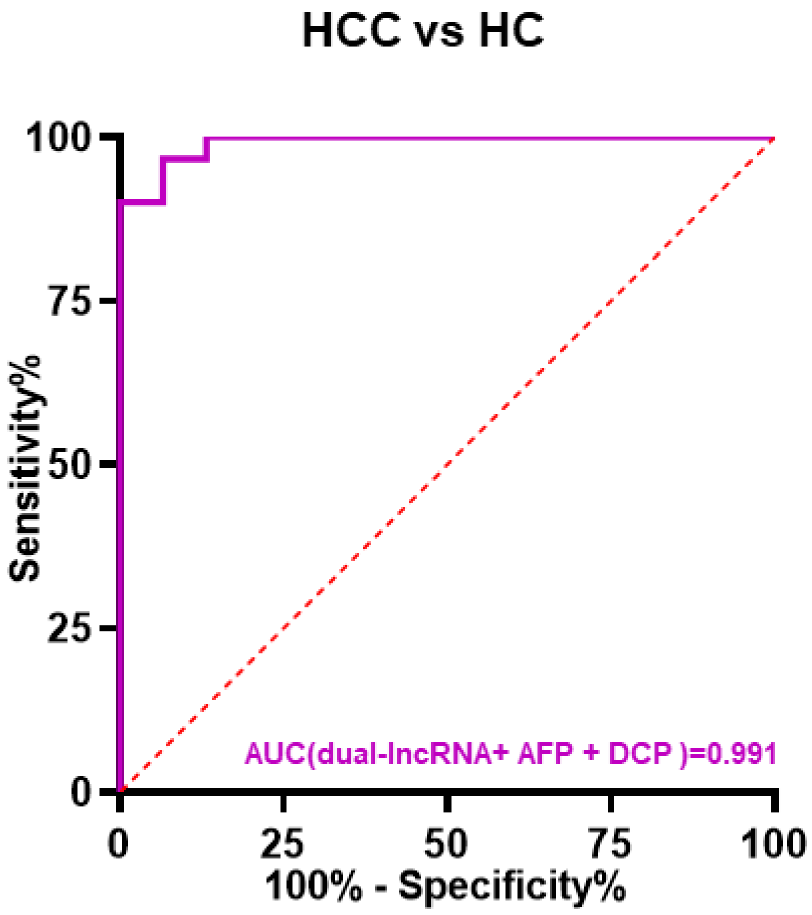


Fig.S7

ROC curve analysis for exosomal dual-lncRNAs + AFP + DCP in the RHWU cohort.
